# Assessment of the Accuracy of Ultrasonographically Measured Optic Nerve Sheath Diameter as a Surrogate for the Detection of Intracranial Hypertension Compared to Optic Nerve Sheath Diameter Measured by MRI: A Prospective Observational Study

**DOI:** 10.7759/cureus.76655

**Published:** 2024-12-30

**Authors:** Seelora Sahu, Nidhi Panda, Amlan Swain, Hemant Bhagat, Shalvi Mahajan

**Affiliations:** 1 Anaesthesiology, Manipal Tata Medical College, Jamshedpur, IND; 2 Anaesthesia, Tata Main Hospital, Jamshedpur, IND; 3 Anaesthesia and Intensive Care, Postgraduate Institute of Medical Education and Research, Chandigarh, IND; 4 Anaesthesiology and Critical Care, Postgraduate Institute of Medical Education and Research, Chandigarh, IND

**Keywords:** intracranial hypertension, magnetic resonance imaging, non-invasive intracranial pressure, optic nerve sheath diameter, ultrasonography

## Abstract

Background: Ultrasonographic measurement of optic nerve sheath diameter (ONSD) has been proposed as a non-invasive, bedside method to detect raised intracranial pressure (ICP) in various clinical settings. We aimed to correlate the ONSD obtained by ultrasonography (USG) with the ONSD obtained by magnetic resonance imaging (MRI) and to find its measurement accuracy.

Methodology: A prospective double-blind study was carried out by performing ocular ultrasounds on 32 patients with clinical features of intracranial hypertension. ONSD was measured by both USG and MRI. Three ultrasonographic scans were performed in both the eyes and the average value of the same was correlated with ONSD obtained from MRI. The three ultrasonographic ONSD scans were also compared among each other for reproducibility and intra-observer variability.

Results: There was a significant correlation between the ONSD measured by MRI and USG (r = 0.954, p = 0.0000, mean difference <5%). A notable degree of agreement between the two measurement modalities was found by the Bland-Altman test (coefficient = -0.116, p = 0.050).

Conclusion: ONSD measured by USG has a significant degree of agreement with that of MRI and hence can serve as an effective, fast, and reliable evaluation technique in patients with intracranial hypertension.

## Introduction

In recent years, there has been a growing interest in measuring the optic nerve sheath diameter (ONSD) as a non-invasive approach for detecting raised intracranial pressure (ICP) [1,2]. ONSD measured by magnetic resonance imaging (MRI) is an effective method of measurement as the scan of the optic nerve sheath gives a clear view, ensuring the diameter is measured with greater accuracy [3]. Additionally, there is no inter-observer variation in the measurement which is a significant shortcoming of ONSD measured by USG [3]. Trans-bulbar ultrasonographic measurement of ONSD has been conducted, and a close association between ONSD and ICP has been found [1]. Numerous studies done in various subsets of patients with dysfunctional intracranial compliance have demonstrated that ONSD is increased in patients with raised ICP; conversely, a reduction in ONSD was witnessed with a decrease in ICP [1-3]. In addition, a decrease or increase in ONSD corresponding to changes in ICP has been demonstrated by studies in the patient population where ICP was artificially manipulated using a blood patch [4,5].

The measurement of ONSD is based on the premise that constant communication exists between the subarachnoid space of the optic nerve sheath and the intracranial cavity [6]. Hence, changes in the ICP could be detected by changes in the diameter of the optic nerve sheath [7]. Expansion of the leptomeningeal sheath of the optic nerve forms the basis of ONSD measurements. This measurement is often taken at a depth of 3 mm from the posterior globe margin, site and is considered to be the most reflective of ICP changes along the long axis of the optic nerve [8-10].

MRI provides great spatial resolution and distinct delineation of soft tissue structures in the body, including the orbital cavity. Alternatively, USG is a bedside, non-invasive, time-efficient, and easily repeatable method for measuring ONSD. A study of healthy volunteers demonstrated the high reproducibility and accuracy of ONSD measurement with USG when compared to MRI [11]. There is very little data in the literature comparing the accuracy of ONSD evaluated by USG vis-à-vis an ONSD obtained by MRI in patients with intracranial hypertension. MRI gives a more accurate value of ONSD as it is measured by an electronic caliper during MRI. Limited access, the need for patient transportation to the MRI suite, space constraints in MRI suites along with high costs are the reasons limiting the regular use of MRI to measure ONSD. In comparison, the measurement of ONSD by USG presents itself as a more straightforward, relatively less expensive, repeatable, bedside technique, making it an attractive proposition to conduct serial ONSD measurements.

We hypothesized that the demonstration of a good correlation between ONSD measured by MRI and USG would provide strong evidence for the acceptance and widespread use of ONSD by USG as a simple and noninvasive bedside monitor of raised ICP. Therefore, we aimed to evaluate the accuracy of the measurement of the trans-bulbar ONSD acquired by USG by establishing a correlation between it and the ONSD obtained by MRI in patients with increased intracranial pressure.

This study abstract was presented at the 52nd Annual Meeting of the Society for Neuroscience in Anaesthesiology and Critical Care and was awarded the 2024 WINNER Abstract Award. The abstract features in the preprint of Volume 37, Journal of Neurosurgical Anaesthesiology (10.1097/ANA.0000000000001013).

## Materials and methods

Study population

This prospective, double-blind observational study included 32 patients presenting with clinical or combined clinical as well as radiological features of raised ICP scheduled for CSF diversion procedures (VP Shunt or ETV) at Nehru Hospital, PGIMER, Chandigarh, India, a leading tertiary care teaching hospital, between July 2013 and Oct 2014. All patients were between 18 and 65 years of age and included individuals of both sexes. The study was carried out after receiving Institutional Ethics Committee approval and written informed consent in accordance with the ethical standards laid down in the Declaration of Helsinki. Patients having (a) orbital pathologies, (b) orbital injuries, (c) disease affecting the optic nerve, and (d) those having glaucoma were not included in the study. The ONSD was measured by USG just before the patient was subjected to an MRI examination as part of their routine diagnostic workup. 

ONSD measured by ultrasonography

The ultrasonographic measurements were performed in the supine position using a 13-6 MHz linear probe (L25e, Sonosite Micromaxx, SonoSite Inc., Bothell, WA), placed over the closed eyelid on the upper temporal side, in accordance with previous studies employing the same modality [7]. The distance between the external borders of the hyperechoic area surrounding the optic nerve, 3 mm behind the globe, was measured by electronic calipers. Three scans were performed in each eye for a particular patient. For each scan, an average of the measurements taken from the left and right eye (six readings) was used to calculate the mean ONSD.

ONSD measured from MRI

All the patients underwent an MRI as a part of the routine diagnostic workup. The ONSD measurements obtained from MRI were carried out using a 3T Magnetom Total Imaging Matrix Trio (Siemens Medical Solutions, Munich, Germany). The axial proton density/T2-weighted turbo spin-echo fat-suppressed sequences were used to measure ONSD from the image slice that provided the best view of the ONSD. The optic nerve sheath was measured 3 mm posterior to the globe using an electronic caliper.

**Figure 1:**
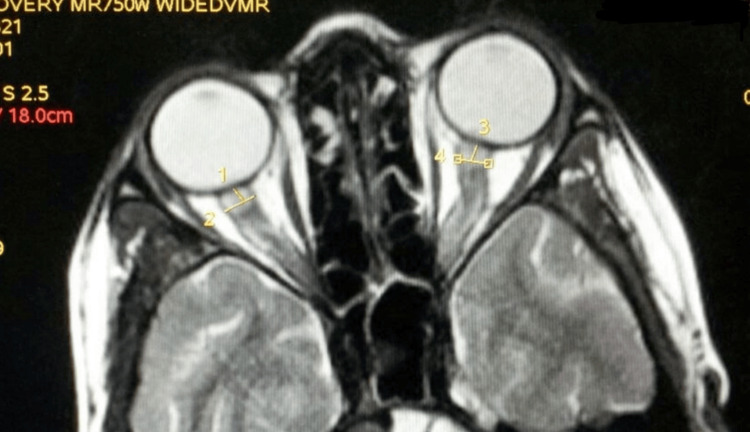
ONSD measurement at 3mm depth by electronic caliper in axial segment of MRI ONSD: optic nerve sheath diameter; MRI: magnetic resonance imaging

The MRI specifications used in our study included: a repetition time of 4,600 ms, echo time of 12 ms, pixel bandwidth of 185 Hz/pixel, 4 mm slice thickness, and 5 mm spacing between slices. The optic nerve sheath on MRI appeared as a zone of high signal surrounding a region of low signal corresponding to the optic nerve. The axial slice providing the best view of the ONSD was selected and the images were magnified to 1,000 × 1,333 pixels using appropriate digital software. The area of interest viz the retrobulbar area was further magnified, subsequent to which ONSD was measured 3 mm behind the globe using an electronic caliper perpendicular to the optic nerve (Figure 1).

The anesthesiologist conducting the USG ONSD measurements was blinded to the ONSD measured by MRI. The radiologist measuring ONSD from MRI film was blinded to the ONSD measured by USG. All the measurements were collated and analyzed by an anesthesiologist who was not involved in any of the ONSD measurements.

Data recorded included ONSD measurements obtained from ultrasonography (Scan 1, Scan 2, Scan 3) and MRIs as well as the demographic variables like age, sex, weight, and American Society of Anaesthesiologists (ASA) physical status, diagnosis, and Glasgow Coma Score (GCS).

Statistical analysis

All the data was analyzed with the aid of SPSS statistical software (version 25, SPSS, Chicago, IL). Parametric data were expressed as mean ± standard deviation. The distribution of the study measurements was verified using the Shapiro-Wilk test. The correlation between the ONSD measured by MRI and the ONSD measured by USG was evaluated using the Bland-Altman test to assess the level of agreement between the two methods and to compare the ultrasonographic technique to the MRI technique. The Bland-Altman test was also used to evaluate the correlation between the three ultrasonographic ONSD measurements to check for reproducibility. The strength of agreement was further quantified using correlation analyses performed by Pearson correlation coefficient (r). Statistical significance was defined at p < 0.05.

## Results

The study was carried out with 32 patients who fulfilled the inclusion and exclusion criteria from among the 58 patients who presented with clinical features of raised ICP. The age of patients ranged from 18 to 65 years with a mean age of 32.73 ± 13.13 years and the mean weight was 59.33 ± 10.35 kg (range 30-80 kg). All patients included in the study had complained of headache while 70% of the patients presented with vomiting (Table 1). All the patients had a GCS of 15. ONSD was measured by USG and MRI in all the patients.

**Table 1:** Demographic parameters GCS: Glasgow Coma Scale, ICP: intracranial pressure, SD: standard deviation, N: number Values are presented as mean ± SD and as *n (%).

Parameters		Values
Age (in years)		32.73 ± 13.13
Weight (in kg)		59.33 ± 10.35
Sex (male: female)		15:17
GCS		14.93 ± 0.25
Duration of presenting symptoms		59.93 ± 56.17
Presenting symptom (number of patients)^*^	Headache	32 (100)
	Vomiting	21(66)
	Vision disturbance	2(7)
	Neurological deficit	3(10)
Aetiology of raised ICP (number of patients)^*^	Obstructive due to mass lesion	24(73)
	Congenital aqueductal stenosis	3(10)
	Aneurysm	4(13.3)
	Infectious	1(3.3)

ONSD was measured by MRI and ultrasonography in both eyes. For ONSD measured by MRI, in a single scan, measurement in each eye was taken and the average was calculated for the analysis. For the ONSD measured by USG, three scans were done in each eye and an average of six measurements was taken for the analysis.

A normal distribution of variables was observed for the ONSD measured by MRI (p = 0.215) and the ONSD measured by USG (p = 0.126). The mean ONSD measured by MRI and USG are tabulated in Table 2.

**Table 2:** The mean ONSD measurements (in mm) by MRI and ultrasonography MRI: magnetic resonance imaging; ONSD: optic nerve sheath diameter; USG: ultrasonography; ONSD: optic nerve sheath diameter; SD: standard deviation Values expressed as mean ± SD.

Parameters	ONSD in mm	Range in mm
MRI	6.21 ± 0.71	4.75 - 7.85
USG (average)	6.28 ± 0.65	4.87 - 7.55
USG Scan 1	6.28 ± 0.63	4.90 - 7.50
USG Scan 2	6.31 ± 0.67	4.65 - 7.60
USG Scan 3	6.25 ± 0.66	4.95 - 7.55

**Figure 2:**
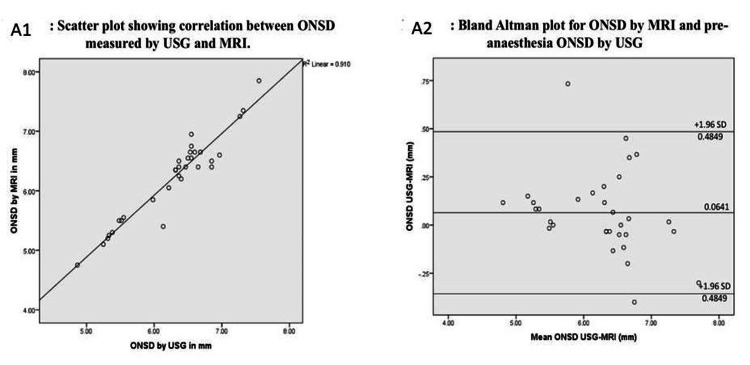
Correlation between ONSD measured by MRI and USG. (A1) Scatter plot; (A2) Bland-Altman plot ONSD: optic nerve sheath diameter; MRI: magnetic resonance imaging; USG: ultrasonography

As summarized in Table 3, there was a significant correlation between the ONSD measured by MRI and the sonographic ONSD (r = 0.954, p = 0.0000) (Figure 2). The Bland-Altman test revealed a good degree of agreement between the two measurement modalities (coefficient = -0.116, p = 0.050) and found a mean difference of 0.061 between the two measurement methods with upper and lower confidence limits ranging from +0.48 to -0.48 (Figure 2).

**Table 3:** Bland-Altman and correlation analysis of ultrasound and MRI ONSD values d: mean difference; σd: limits of agreement; r: correlation coefficient; ONSD: optic nerve sheath diameter

Parameters	Correlation		Reproducibility (Bland-Altman test)			
ONSD	r	p	d	σd	Coefficient	p
MRI - USG	0.954	0.000	0.072	± 0.44	-0.116	0.050

**Figure 3:**
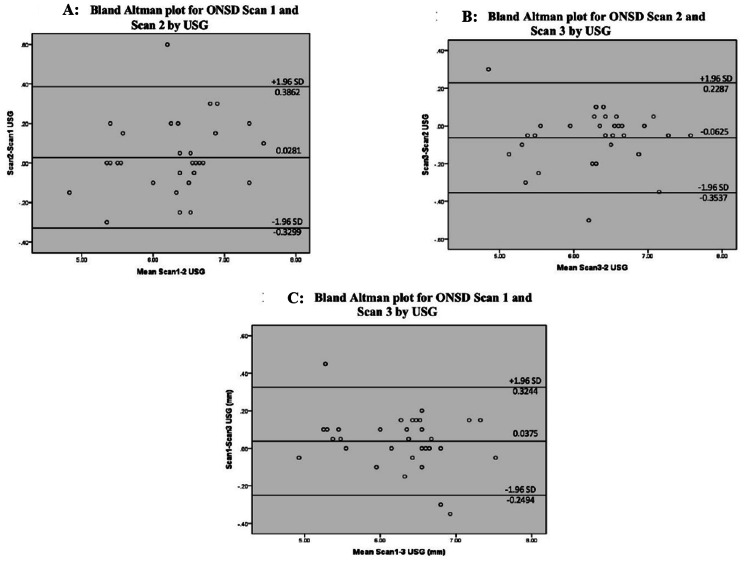
Bland Altman plot correlation between the three ultrasonographic ONSD measurements to check for reproducibility. (A) Bland-Altman plot correlation between USG scan 1 and 2, (B) Bland-Altman plot correlation between USG scan 2 and 3 and (C) Bland-Altman plot correlation between USG scan 1 and 3.

The intra-observer reproducibility of measurements as evaluated by Pearson’s correlation and Bland-Altman revealed a good level of agreement between the three scans done to measure ONSD by transbulbar ultrasonography (Figure 3, Table 4).

**Table 4:** Bland-Altman and correlation analysis of the three ultrasonographic ONSD values d: mean difference; σd: limits of agreement; r: correlation coefficient; ONSD: optic nerve sheath diameter

Parameters	Correlation		Reproducibility (Bland-Altman test)			
ONSD Scan	r	p	d	σd	Coefficient	p
Scan 1 - Scan 2	0.962	0	0.028	± 0.36	0.055	0.325
Scan 2 - Scan 3	0.976	0	-0.063	± 0.28	-0.005	0.912
Scan 3 - Scan 1	0.976	0	0.037	± 0.28	-0.056	0.134

## Discussion

In our study, we observed high measurement accuracy of ultrasonographic ONSD measurements, as evaluated by comparison with the ONSD measured by MRI in patients with intracranial hypertension.

We used ONSD measured from MRI as a standard against which ONSD by USG was compared. MRI is an imaging modality offering high spatial resolution and clear delineation of soft tissue structures. High reproducibility for the MRI-based measurement of ONSD was first reported in a study on Thiel-fixated cadaver specimens [12]. Another study by Lagrèze et al. found that the MRI was more precise for determining the optic nerve and nerve sheath diameter. In their study, they took the measurements at a depth of 5 mm posterior to the globe and not at 3 mm [13]. This was followed by another study in which the authors found that there was a significant correlation of measurement between MRI and high-resolution USG [14].

Later, a study done by Bäuerle et al. in 2013 compared sonographic ONSD measurements with MRI ONSD measurements in healthy individuals [11]. They reported that USG ONSD measures performed at a depth of 3 mm from the optic nerve papilla are more precise and correlate with MRI ONSD measurements at the same depth. In our study, all the ONSD measurements were performed 3 mm posterior to the globe's posterior edge. Our study population included patients with intracranial hypertension while the aforementioned authors had assessed healthy volunteers in their study.

In a study by Steinborn et al., the mean ONSD values were found to be in the range of 5.86 ± 0.66 mm and 5.86 ± 0.71 mm by MRI and USG respectively in children with variable neurological pathologies [14]. Comparatively, the mean ONSD values in our study were MRI = 6.21 ± 0.71 mm, and USG = 6.28 ± 0.65 mm. A possible explanation for this discrepancy can be the fact that the former study was conducted in children (3 months to 11 years), representing developmentally smaller anatomical values. Similarly, another study by Bäuerle et al. found the means ONSD value on MRI = 5.69 ± 0.77 mm, USG = 5.43 ± 0.49 mm [11]. This study was done in healthy volunteers who had normal intra-cranial pressure while our study included patients with raised ICP; this could account for the higher ONSD values in our study.

Our study reports a significant agreement between the ONSD measured by USG and that determined by MRI, with readings differing by less than 5%. In our study analysis, the boundaries of an agreement are narrower than the study by Steinborn et al [14]. The smaller margins of agreement can be explained by the selection of anatomically developed adult patients in our study cohort, which resulted in less variation in standard anatomical measurements. A high degree of agreement between the USG and MRI values of ONSD was also reported in healthy individuals by studies by Bäuerle et al. and Ballantyne et al [15,16]. Another study conducted on adult patients with diagnosed meningoencephalitis admitted to the intensive care unit also found an acceptable agreement between the ONSD calculated using ultrasonography with ONSD measured by MRI [17].

Limitations

One of the limitations was the single-center design study with a relatively small group of patients. Additionally, we did not compare the ONSD measurements obtained from patients with elevated ICP with controls without intracranial hypertension.

The relatively large slice thickness and interslice spacing (4 mm and 5 mm, respectively) was another study limitation, related to the technical ramifications of MRI. MRI protocols employing thinner slices or three-dimensional volumetric acquisition would probably increase the precision of ONSD measurements. The two measurements of ONSD (ultrasonography and MRI) were not performed simultaneously, and it has been demonstrated that ONSD fluctuates rapidly in reaction to stress or stimulus [18]. However, this technical constraint will persist until MRI-compatible ultrasonography devices are available. In the coming years, AI-based automation of ONSD measurements acquired by MRI or ultrasonography can be further explored to enhance the diagnostic accuracy similar to the other diagnostic and therapeutic advances in these subsets of patients [19].

## Conclusions

In patients with elevated intracranial pressure, ultrasonographic ONSD measurement can be performed with accuracy, robust reproducibility, and good intra-observer agreement. It has a strong correlation with ONSD measured from T2-weighted MRI and can be used as a surrogate measure of the ICP. Thus the sonographic method for measuring ONSD offers a cost-effective and reliable bedside method to detect intracranial pressure that can quantify information with accuracy similar to MRI.
